# The Breadth of Cross Sub-Type Neutralisation Activity of a Single Domain Antibody to Influenza Hemagglutinin Can Be Increased by Antibody Valency

**DOI:** 10.1371/journal.pone.0103294

**Published:** 2014-08-01

**Authors:** Simon E. Hufton, Paul Risley, Christina R. Ball, Diane Major, Othmar G. Engelhardt, Stephen Poole

**Affiliations:** 1 Biotherapeutics Group, National Institute for Biological Standards and Control, a centre of the Medicines and Healthcare Products Regulatory Agency, South Mimms, Potters Bar, Hertfordshire, United Kingdom; 2 Division of Virology, National Institute for Biological Standards and Control, a centre of the Medicines and Healthcare Products Regulatory Agency, South Mimms, Potters Bar, Hertfordshire, United Kingdom; 3 Technology Development and Infrastructure, National Institute for Biological Standards and Control, a centre of the Medicines and Healthcare Products Regulatory Agency, South Mimms, Potters Bar, Hertfordshire, United Kingdom; German Primate Center, Germany

## Abstract

The response to the 2009 A(H1N1) influenza pandemic has highlighted the need for additional strategies for intervention which preclude the prior availability of the influenza strain. Here, 18 single domain VHH antibodies against the 2009 A(H1N1) hemagglutinin (HA) have been isolated from a immune alpaca phage displayed library. These antibodies have been grouped as having either (i) non-neutralising, (ii) H1N1 restricted neutralising or (iii) broad cross-subtype neutralising activity. The ability to neutralise different viral subtypes, including highly pathogenic avian influenza (H5N1), correlated with the absence of hemagglutination inhibition activity, loss of binding to HA at acid pH and the absence of binding to the head domain containing the receptor binding site. This data supports their binding to epitopes in the HA stem region and a mechanism of action other than blocking viral attachment to cell surface receptors. After conversion of cross-neutralising antibodies R1a-B6 and R1a-A5 into a bivalent format, no significant enhancement in neutralisation activity was seen against A(H1N1) and A(H5N1) viruses. However, bivalent R1a-B6 showed an 18 fold enhancement in potency against A(H9N2) virus and, surprisingly, gained the ability to neutralise an A(H2N2) virus. This demonstrates that cross-neutralising antibodies, which make lower affinity interactions with the membrane proximal stem region of more divergent HA sub-types, can be optimised by bivalency so increasing their breadth of anti-viral activity. The broad neutralising activity and favourable characteristics, such as high stability, simple engineering into bivalent molecules and low cost production make these single domain antibodies attractive candidates for diagnostics and immunotherapy of pandemic influenza.

## Introduction

Pandemic influenza generally occurs when a new virus emerges and infects the global human population which has little or no pre-existing immunity [1]. The most recent H1N1 pandemic in 2009, although a considerable economic burden, fortunately did not result in the same rates of mortality as has been seen for previous pandemics [2], [3]. Of continuing concern is highly pathogenic avian influenza (HPAI) which has demonstrated mortality rates of greater than 50% in infected humans [4]. H5 virus is endemic in poultry in certain parts of the world and currently does not appear to be able to transmit readily from person to person despite causing at least 384 deaths worldwide (WHO website, http://who.int/influenza/human_animal_interface/EN_GIP_20131210CumulativeNumber H5N1 cases.pdf.accessed14Jan2014). However, recent data confirm that very few amino acid changes (approximately 5) are required to enable this avian virus to spread through aerosol transmission in a mammalian *in vivo* model system [5], [6].

Although vaccines are the main method of infection control, their timely implementation presents several technical challenges. These include (i) prediction of which viral strains will emerge and infect the human population, (ii) the lag period between the appearance of a new viral strain and the availability of a clinically approved vaccine, (iii) poor immunogenicity in certain patient groups, for example the elderly, very young or immune-compromised (iv) limited worldwide production capacity. Anti-viral drugs such as oseltamavir and rimantadine are an important addition to the arsenal of treatment options against both seasonal and pandemic influenza, however, resistance has been observed and they will inevitably become ineffective over time [7], [8]. There is clearly a need for other treatments and the concept of a ‘universal therapy’ which overcomes the virus's ability to alter its viral coat structure and evade immune detection is receiving renewed interest [9]–[11]. Antibodies represent one of the earliest classes of protective agents and the passive transfer of serum from convalescent patients was used during the 1918 pandemic [12] and more recently to treat a severely ill H5N1 patient [13]. However, this approach has limited potential for implementation on a global scale due to restricted supply of appropriate sera, high risk of toxicity, high lot-to-lot variation, uncertain dosing and difficulties in administration. Recent advances in recombinant monoclonal antibody technology have made this strategy worthy of further investigation, not least because large quantities of protective antibodies can be stock-piled to provide immediate protection in a pandemic emergency [10], [14]. For this to be an effective strategy such antibodies would be required to have neutralising reactivity across different viral sub-types. This presents a challenge as the influenza virus is constantly changing which means that immunity through exposure to one viral strain does not necessarily provide adequate protection against future strains. Yet the existence of individuals who have immunity to strains to which they have not been previously exposed suggests that cross-protective immunity is possible [15]. It has been proposed that certain structures of the influenza virus coat proteins, such as the hemagglutinin (HA) stem region [16]–[23], the extracellular domain of the M2 ion channel [24], [25] and neuraminidase [26] as well as highly conserved internal nucleoprotein (NP) [27] are the targets for this cross-protective immunity. Hemagglutinin (HA) is the major viral coat protein and mediates binding to cell surface sialic acid, so initiating virus infection [28]. Sequence analysis reveals that there is considerable variation in the HA gene which subdivides influenza strains into 4 different clades within two phylogenetic groups; group 1 which contains 10 of the 16 subtypes including the H1 clade (H1, H2, H5, H6, H11, H13 and H16) and H9 clade (H8, H9, and H12), and group 2, which includes the H3 clade (H3, H4, and H14) and the H7 clade (H7, H10, H15) [29]–[31]. Each of these two phylogenetic groups has a highly variable globular head region (HA1) which mediates sialic acid receptor binding and a more conserved proximal stem region which is principally comprised of the HA2 domain and some of the HA1 domain [28]. Until recently, monoclonal antibodies that show broad neutralisation activity across different influenza subtypes have been scarce with most antibodies being directed to the variable globular head domain (HA1). The recent isolation of cross-neutralising human monoclonal antibodies to the more conserved HA stem region [16]–[23], [32] was surprising and questions why has it been so difficult to identify this type of antibody in the past. The answer may, at least partially, be in the virus striving to keep its most important and conserved determinants of pathogenicity hidden, combined with the challenges the human immune system has in accessing these parts of the viral coat structure. Antibodies to these conserved epitopes are likely to occur rarely and it is only through the advent of sophisticated antibody engineering techniques such as phage display that it has become easier to isolate such monoclonal antibodies. Two recent examples are the human monoclonal antibodies F10 [16] and CR6261 [17] which have both been shown to bind to a highly conserved binding pocket in the membrane proximal stem region using only their heavy chain for antigen binding, with no antigen contact being made by their light chains. This suggests that ‘heavy-chain only’ recognition may be a preferred mode of binding for broadly neutralising antibodies to influenza as has been suggested for cross-neutralising antibodies to HIV [33], [34]. Furthermore, both F10 and CR6261 use the human germline segment VH 1-69 with very little somatic hypermutation which suggests that these two antibodies may be the product of an immediate and essentially germline response to the virus [16], [17], [35], [36]. These observations imply that the VL domain may not be required in accessing these important viral epitopes and may actually be a hindrance. The existence of naturally occurring ‘heavy chain only’ antibodies is well documented in camelid species [37], [38] and their unique properties are being exploited for wide ranging applications in biotechnology including immunotherapy [39], [40]. These single domain antibodies (sdAbs), also called Nanobodies (Ablynx N.V) have several advantages over conventional monoclonal antibodies including; (i) small size (15 kDa), (ii) low cost microbiological production, (iii) simple engineering into bi-specific formats, (iv) high stability with the potential to support non-injectable routes of administration, and (iv) potential to access buried or hidden epitopes through long CDR3 loops [40]. In the present study, we have chosen to exploit the unique structural features of single domain antibodies from camelids, which are naturally free of a paired light chain, as a route to high affinity, robust antibodies to neutralising epitopes on influenza HA. We have set out to isolate sdAbs capable of neutralising influenza viruses across divergent viral subtypes particularly the recent 2009 pandemic A(H1N1) and highly pathogenic avian influenza A(H5N1). To achieve this objective an immune alpaca phage displayed single domain VHH antibody library was constructed and selected with both recombinant H1 and H5 antigens. The resultant antibodies were characterised for their binding specificity, ability to neutralise different viral sub-types and the location of their antibody epitopes. For two of the most potent cross-neutralising antibodies, we investigated to what extent antibody valency can enhance viral neutralisation activity. These findings and the potential applications of these antibodies are discussed.

## Experimental Procedures

### Influenza antigens and immunisation of alpacas

The virus antigen standards used in this study were derived from A/California/07/2009 (H1N1)pdm09, A/Brisbane/59/2007 (seasonal H1N1), A/Indonesia/05/2005 (H5N1); A/Vietnam/1194/2004(H5N1), A/Anhui/01/2005(H5N1), A/turkey/Turkey/01/2005(H5N1), A/HongKong/213/2003(H5N1), A/duck/Singapore/Q/F119-3/97(H5N3), A/Singapore/01/57(H2N2), A/mallard/England/727/2006(H2N3), A/HongKong/1073/99(H9N2), A/Brisbane/10/2007(H3N2) and B/Brisbane/60/2008-like (National Institute for Biological Standards and Control). Purified recombinant hemagglutinins used in this study were H1 (A/California/07/2009), H5(A/Vietnam/1203/2004), H9(A/HongKong/1073/99), H7(A/Netherlands/219/2003), H3(A/Brisbane/10/2007)(ProteinSciences) and H1 (D18-I530)(A/California/06/2009), HA1 domain (D18-R344)(A/California/06/2009) and H2 (A/Japan/305/57) (E-Enzymes). The laboratory-adapted strains X-181, a conventional reassortant virus containing the HA and NA genes of A/California/07/2009 (H1N1)pdm09, NIBRG-14 a reverse genetics reassortant of A/Vietnam/1194/2004 (H5N1) with the polybasic cleavage site removed from the HA, NIBRG-147 a reverse genetics reassortant of A/Singapore/01/57 (H2N2), NIBRG-91, a reverse genetics reassortant of A/chicken/Hong Kong/G9/97 (H9N2) and NIBRG-109, a reverse genetics reassortant of A/New York/107/2003 (H7N2) (National Institute for Biological Standards and Control) were used in viral neutralisation assays [41].

Juvenile male alpacas were purchased through the Royal Veterinary College, Hertfordshire, UK. All experiments were reviewed by the NIBSC (National Institute for Biological Standards and Control) local ethics committee and performed under a U.K Home Office licence. A blood sample prior to immunisation was obtained from the external jugular vein and this was followed by 4 intramuscular injections on day 0 (primary immunisation), 21, 43 and 71 with injections being made in the rear legs (thigh region) on days 0 and 43 and in front legs (thigh region) on days 21 and 71. The primary immunisation consisted of 50 µg of recombinant HA (A/California/07/2009) (H1N1)pdm09 (Protein Sciences) in 400 µl of sterile PBS and emulsified with 800 µl of complete Freund's adjuvant (Sigma) just prior to immunisation. Similarly, three separate booster injections of 50 µg of recombinant H1-HA in incomplete Freund's adjuvant (Sigma) were administered. Approximately four days after each injection, a 10 ml blood sample was collected from the external jugular vein from which serum was prepared after allowing the blood to clot overnight at 4°C.

### Construction and selection of phage displayed libraries

For antibody library construction approximately 10 ml samples of blood were collected from an immunised alpaca into heparinised tubes. Peripheral blood lymphocytes were purified using a ficol hypaque centrifugation procedure (Sigma) and RNA was extracted using a RiboPure RNA extraction kit (Novagen) according to manufacturer's instructions. First strand cDNA synthesis was performed using Superscript III reverse transcriptase (Invitrogen) and oligo-dT primer with 200 ng of total RNA per reaction. Primary PCR was performed using oligonucleotide primers designed to universally prime mammalian immunoglobulin genes in the CH2 domain and also at the 5′ end of alpaca VHH genes within the signal sequence [37], [42], [42,43] [Alp-CH2_Rev, 5′ CGC CAT CAA GGT ACC AGT TGA: AlpL-Fw_VHH, 5′ GGT GGT CCT GGC TGC]. High fidelity taq polymerase (Roche) was used for all DNA amplifications. A product of approximately 600 bp corresponding to the heavy chain only antibody population was gel purified (QIAGEN) and then subjected to a secondary PCR step to amplify only the VHH antibody gene population (∼450 bp) and to append appropriate restriction sites for cloning into the phage display vector pNIBS-1 [the phage display vector pNIBS-1 was constructed in this study by cloning a polylinker comprising a pelB signal sequence, *Sfi*1 and *Not*1 restrictions sites, His-tag, c-myc tag, amber stop codon, gene III protein into pUC119 phagemid vector]. Primers for secondary PCR were Alp_FR1_*Sfi*1 - 5′ CTG CAG GGA TCC GTT AGC AAG GCC CAG CCG GCC ATG GCA CAG KTG CAG CTC GTG GAG TCN GGN GG; Alp_FR4back_*Not*1 - 5′ GCT AGT GCA TGG AGC TCA TGC GGC CGC TGA GGA GAC GGT GAC CTG. Approximately 5 µg of VHH antibody DNA was digested with *Sfi*I restriction enzyme (New England Biolabs) in a 200 µl reaction overnight at 50°C. After further purification (QIAGEN) the VHH genes were digested with *Not*1 in a 100 µl reaction at 37°C for 6 hours. The digested VHH genes were then ligated into the phage display vector pNIBS-1 which was similarly digested with *Sfi*1 and *Not*1. After purification, the ligation mix was transformed into TG1 electro-competent cells (Agilent) using electroporation (BIO-RAD). Transformants were spread on 22 cm bioassay dishes (Corning) containing TY agar supplemented with carbenicillin (100 µg/ml) and 20% (w/v) glucose. The library size was 2.4×10^8^ independent clones and was harvested using standard procedure [44], [45].

Phage antibody library selections were performed essentially as in [44], [45] using immunotubes (Nunc) coated overnight at 4°C with 1 ml of 10 µg/ml recombinant HA (Protein Sciences) in PBS. Bound phage antibodies were eluted by adding 1 ml of 100 mM triethylamine followed by incubation for 10 minutes on a rotating platform at room temperature. The eluted phage were neutralized with 0.5 ml 1 M Tris-HCl pH 7.5. To amplify the selected phage for subsequent rounds of selection 1 ml of eluted phage were mixed with 5 ml of an *Escherichia coli* ER2738 (Agilent) culture grown to an OD_600_ of 0.5 and 4 ml of 2 × TY media followed by incubation in a water bath at 37°C for 30 minutes. This was then spread onto 22 cm bioassay dishes containing 2 × TY agar supplemented with 100 µg/ml (w/v) carbenicillin and 2% (w/v) glucose. Plates were grown overnight at 37°C and harvested and the phage titres before and after selection were determined [44], [45].

To construct a gene fragment library, full length hemagglutinin gene of 1704 bp from A/California/07/2009 (H1N1)pdm09 was synthesised from overlapping oligonucleotides and sequence verified (IDT Technologies). The phage display vector pNIBS-1 was modified so as to incorporate a *Sma*1 blunt end restriction site to create pNIBS-blunt. Using blunt end cloning one out of nine randomly generated gene fragments was predicted to be in the correct reading frame at the N- and C- terminus for potential display on the surface of filamentous M13 phage. Purified DNA corresponding to full length HA gene was digested with DNAase using a shotgun cleavage kit (Novagen) according to the manufacturer's instructions to obtain DNA fragments in the size ranges of 50–1000 bp. The gene fragments were converted into blunt ended fragments as per manufacturer's instructions and cloned into pNIBS-blunt digested with *Sma*1 and treated with alkaline phosphatase. After purification of the ligation mix the cloned DNA was transformed by electroporation (BIO-RAD) into electro-competent *Escherichia coli* TG1 cells (Agilent) and spread onto large 22 cm dishes containing TY agar supplemented with carbenicillin 100 µg/ml (w/v) and 2% (w/v) glucose. A library of 50 bp–1 kb fragment range, and size 8×10^6^ independent clones was generated with >95% inserts after sequence analysis of 30 randomly chosen clones (data not shown). The library was rescued and selected using immunotubes coated with purified VHH antibodies at 10 µg/ml in PBS or serum from sheep hyper-immunised with HA of A/California/07/2009 (H1N1)pdm09 (National Institute for Biological Standards and Control). In parallel a blank immunotube containing PBS was used to monitor the selection process. Clones were randomly picked and sequenced. Phage corresponding to the HA gene fragments identified were purified using polyethylene glycol precipitation and sterile filtration through a 0.2 µm filter (GE Healthcare). Selected phage HA clones were then tested for binding to purified VHH antibodies using ELISA.

### Antibody expression and screening

Primary screening was carried out using soluble VHH antibodies harvested from the culture supernatant in a 96 well format. In short, individual colonies from each round of selection were inoculated into 100 µl of 2 × TY medium supplemented with 100 µg/ml (w/v) carbenicillin and 2% (w/v) glucose in a 96 well flat bottom plate (Costar) using sterile toothpicks and grown overnight in a shaking incubator at 30°C. From this master plate a new 96 well round bottom plate (Costar) containing 120 µl of 2 × TY supplemented with carbenicillin (100 µg/ml) and 0.1% (w/v) glucose was inoculated and grown for 6 hours at 37°C until OD_600_ of approximately 0.9 was reached, after which time 30 µl of 2 × TY supplemented with 100 µg/ml carbenicillin plus 5 mM IPTG (1 mM final concentration) was added with continued shaking at 37°C overnight. The plates were then centrifuged at 600 × g for 10 minutes and the supernatant containing soluble VHH antibodies was used to test antigen-specific reactivity to HA using ELISA. The ELISA was performed in a 96 well plate (Nunc) coated with recombinant HA at 1 µg/ml overnight in PBS at 4°C. To detect antibody binding 100 µl of anti c-myc 9E10-HRP (1/1000 dilution) (Roche) in 2% (w/v) milk powder in PBS was added and developed using standard methods. For their large scale production VHH antibodies were transformed into the non-suppressor *Escherichia coli* strain WK6 (New England Biolabs). Soluble VHH antibodies were purified from either 50 ml cultures or 500 ml cultures [45], [46]. Soluble antibody expression was induced with the addition of 1 mM IPTG followed by further incubation overnight at 30°C. Periplasmic extracts were prepared as in [46] and were dialysed using cassettes with a 3 kDa molecular weight cut off (Pierce) into PBS with two changes of buffer. Antibody was purified by immobilised metal chelate chromatography (IMAC) using Talon resin according to manufacturer's instructions (Clontech). Antibodies were further purified by size exclusion chromatography on a Superdex 75 10/300 GL column (GE Healthcare) run in PBS and pooled fractions were sterile filtered using millex–GV filter units (Millex). The size and purity of bivalent and corresponding monovalent antibodies was assessed using analytical SE-HPLC and SDS-PAGE. Influenza virus antigen standards (National Institute for Biological Standards and Control) were reconstituted in 1 ml sterile water and then diluted 1/200 in 0.5 M bicarbonate buffer pH 9.6 prior to incubation overnight at 4°C in a 96 well plate (Nunc). Plates were washed and processed as described above except a serial dilution of purified VHH antibodies was used of 30, 10, 3, 1, 0.3, 0.1, 0.03, 0.01, 0.003, 0.001, 0.0003, 0.0001 µg/ml.

Low pH-induced conformational change in HA was performed as below. An antigen standard of H1N1 [NIBSC 09/146 (A/California/07/2009)H1N1pdm09] was re-suspended in 1 ml of PBS and 100 µl was diluted to 20 ml in PBS and the pH reduced to approximately pH 3.0 with 1.5 ml of 1 M hydrochloric acid. After incubation for 2.5 hours at room temperature, the antigen was neutralised with 20 mls of 0.5 M bicarbonate buffer pH 9.6 to give a final pH of approximately 9.0. This acid-treated antigen was then used to coat ELISA plates overnight at 4°C. The next day ELISA plates were blocked with 2% MPBS and antibody binding was assessed as above using anti c-myc-HRP (1/1000 dilution) (Roche) in 2% (w/v) milk powder in PBS. A parallel assay was conducted using H1N1 antigen prepared in exactly the same way except treatment with acid was omitted.

### Hemagglutination inhibition (HI) and microneutralisation (MN) assays

The hemagglutination inhibition (HI) assay was performed using serial dilutions of serum from immunized alpacas or purified VHH antibodies starting from 250 µg/ml. Serial dilutions of antibody were incubated with four HA units of virus per well followed by the addition of turkey erythrocytes to a concentration of 50% (v/v). The plate was incubated at room temperature for 30 minutes and the hemagglutination inhibition (HI) titre was scored as the reciprocal of the last antibody dilution that completely inhibited hemagglutination.

The influenza microneutralisation assay (MN) was performed essentially as in [47]. Initially the tissue culture 50% infectious dose (TCID_50_) of virus was determined using the Reed Muench method. Standard overnight cell culture endpoint dilution assays were performed on the reverse genetics reassortant attenuated influenza viruses NIBRG-14 (A/Vietnam/1194/2004; H5N1), X-181 (A/California/07/2009; H1N1), NIBRG-147 (A/Singapore/1/57; H2N2), NIBRG-91 (A/chicken/HongKong/G9/97; H9N2) and NIBRG-109 (A/New York/107/2003 ; H7N7). Viruses were diluted to two log values above the TCID_50_. A two fold serial dilution of either serum from immunised alpacas or purified VHH antibodies (starting concentration of 4 µM) was prepared in a 50 µl volume of assay diluent [DMEM with the addition of 4 mM glutamine, penicillin and streptomycin (Sigma) 1/100 (v/v), amphotericin 2.5 µg/ml, non-essential amino acids 1/100 (v/v) 20 mM HEPES and fetal calf serum 1/100 (v/v)] and added to a 96 well flat bottom plate (Becton Dickenson). Fifty microlitres of virus, diluted in assay diluent was then added to each well. Controls containing only virus (no antibody) and cells only (no virus) were included on each assay plate. Plates were incubated at room temperature for 1 hour after which 100 µl of 1.5×10^5^/ml MDCK cells were added to all wells and incubated for 18 hours at 37°C in 5% CO_2_. Plates were aspirated and fixed by adding 200 µl of methanol/hydrogen peroxide 0.6% (v/v) to all wells for 20 minutes at room temperature. The plate wells were checked for cell growth and then washed 3 times in PBS/Tween-20 (0.05% v/v)(PBST). Plates were blocked for 1 hour with PBS/Tween-20 0.1%(v/v) and 2% (w/v) milk powder, (MPBST), followed by the addition of 100 µl of mouse anti-influenza A nucleoprotein (ABD Serotec) (1∶3000 dilution in MPBST) to all wells and incubated for 1 hour at 37°C. Plates were washed 3 times with PBS and 100 µl of secondary antibody, rabbit anti-mouse IgG polyclonal HRP conjugate (DAKO), (1∶5000 MPBST) and 100 µl was added to each well and incubated for 1 hour at 37°C. Plates were washed 4 times with PBST and the ELISA was developed using standard TMB staining methods. The antibody concentration that represented 50% virus neutralisation was determined [48] and values were given either as the reciprocal of the serum dilution or the antibody concentration in nM. Micro-neutralisation assays using purified bivalent antibodies were performed using molar equivalent amounts of monovalent and bivalent versions of R1a-A5 and R1a-B6.

### Analysis using surface plasmon resonance

For binding and affinity ranking a BIAcore T100 machine (GE Healthcare) was used in combination with a single cycle kinetics procedure [49]. In brief, purified recombinant hemagglutinins of different subtypes (H1, A/California/04/2009; H5, A/Vietnam/1203/2004; H9, A/HongKong/1073/99; H7, A/Netherlands/219/2003; H3, A/Brisbane/10/2007; H2, A/Japan/305/57) (Protein Sciences) were immobilised onto a BIAcore CM5 chip in 10 mM sodium acetate pH 5.5 using an amine coupling kit (GE Healthcare) to approximately 3,000 RU or 10,000 RU for a high density surface. A concentration series of purified sdAbs was sequentially run over the different antigen surfaces [49]. A reference surface was subtracted prior to evaluation of the sensorgrams using the single cycle kinetics procedure of BIAevaluation software (GE Healthcare) and a 1∶1 fitting model. Binding to the globular head of hemagglutinin was evaluated using recombinant HA1 domain (D18-R344) (A/California/06/2009) (H1N1)pdm09 and full length HA (D18-I530) (E-Enzymes) immobilised on a CM5 chip in combination with a single cycle kinetics procedure as described above.

SPR co-injection procedures were used to evaluate whether or not antibodies recognised unique or overlapping epitopes. In brief, the first VHH antibody diluted in HBS-EP+ buffer (GE Healthcare) to 100 nM was injected over a surface of recombinant H1 (approximately 10,000 RU) at 30 µl/min for 500 seconds. Following injection of the first VHH antibody either buffer or a second VHH antibody (100 nM at 30 µl/min) was injected over the surface already bound with the first VHH antibody for 200 seconds. The antibodies R1a-A5, R1a-B6, R2b-E8, R2b-D9, R2a-G8, R1a-C5 and R2a-G9 were tested. Antibodies predicted to share overlapping epitopes were further tested by comparing the maximum binding levels (Rmax) reached using equimolar mixes of antibodies with those reached by antibodies tested individually at the same concentration.

Similar SPR co-injection experiments were used to evaluate competition with the cross- neutralising mouse monoclonal antibody C179 (Takara Bio Inc) [50], [51] which recognises a conformation specific epitope in the HA stem region. Antibody C179 was reconstituted according to manufacturer's instruction and diluted to 50 nM in HBS-EP+ (GE Healthcare). C179 was injected over a surface of recombinant H1 (approximately 10,000 RU) at 30 µl/min for 500 seconds. Following injection of C179 antibody either buffer or the cross reactive sdAbs R1a-A5, R1a-B6, R2b-D9, R2b-E8, R2a-G8 (100 nM at 30 µl/min) were injected for 200 seconds over the surface already bound with C179.

### Cloning and purification of bivalent antibodies

Antibodies R1a-B6 and R1a-A5 were converted into bivalent molecules using a (G4S)6 linker to fuse together two identical VHH domains. Sequences of each VHH antibody unit were designed to limit the percentage of GC content and to reduce internal homology within the construct. Bivalent genes were assembled by PCR from overlapping oligonucleotides (IDT technologies). Bivalent antibodies were sub-cloned into pNIBS-1 and transformed into the *Escherichia coli* strain WK6. Antibodies were expressed and purified as for the monovalent VHH antibodies using immobilised metal chelate chromatography from the periplasmic fraction using Talon resin (Clontech). Eluted antibodies were dialysed against PBS using dialysis cassettes of molecular weight cut off of 3 kDa (Pierce).

## Results

### Isolation of H1 and H5 specific single domain antibodies

A juvenile male alpaca was immunized with recombinant HA derived from the recent pandemic A(H1N1) strain (A/California/07/2009)(H1N1pdm09)[52]. The immune response was evaluated using ELISA (data not shown), hemagglutination inhibition assay (HI assay) and microneutralisation (MN) assay with A/California/07/2009 (H1N1)pdm09, as well as HI with viral strains representative of highly pathogenic A(H5N1). A clear serological immune response against pandemic H1N1 virus was seen by both HI and MN assay (Table S1).

**Figure 1:**
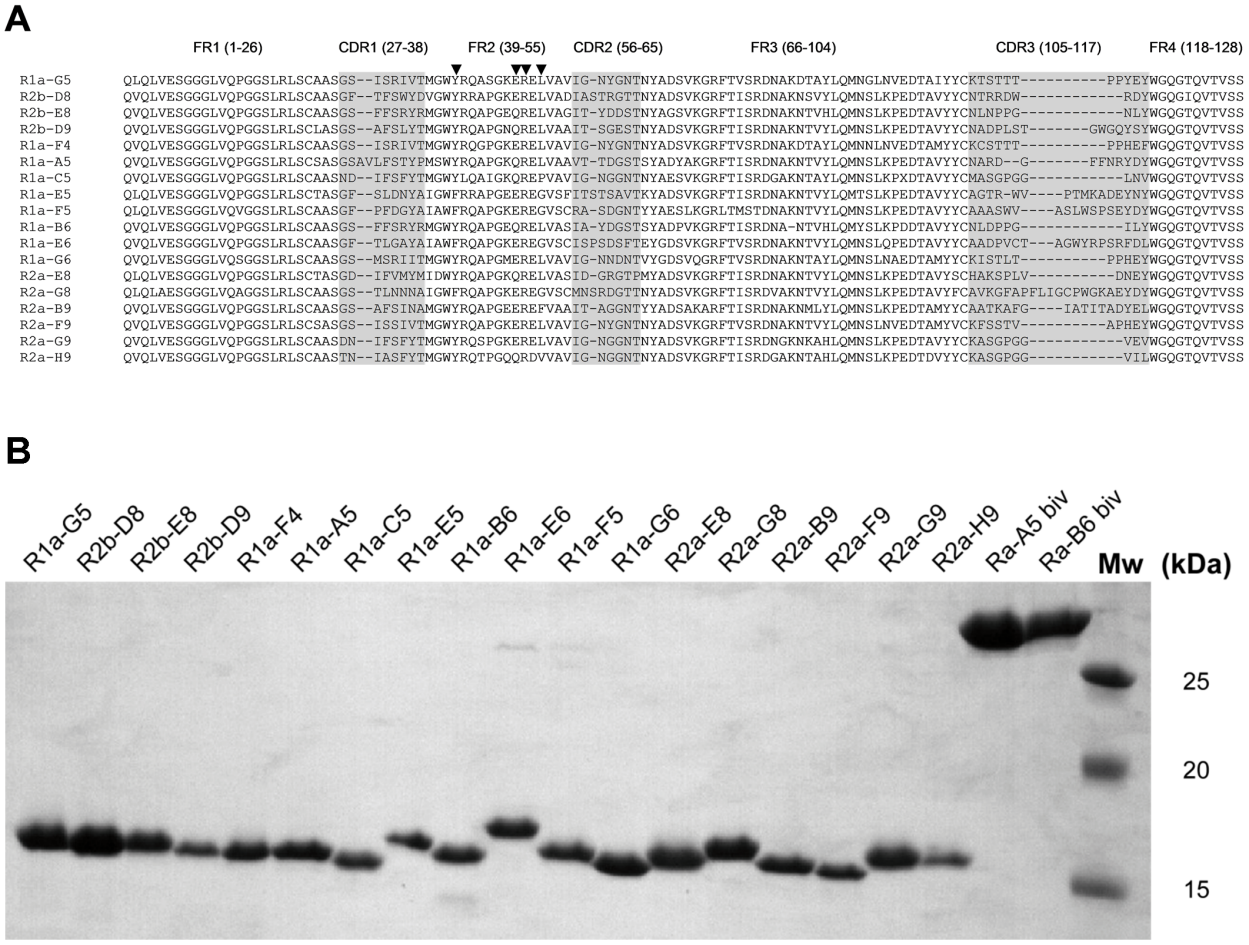
Sequence analysis and purification of selected single domain antibodies. (A) Sequence alignment of unique single domain VHH antibodies specific to pandemic H1N1 isolated in this study. CDR and framework regions are defined according to the IMGT numbering scheme (http://imgt.cines.fr/). The hallmark residues in framework region 2 (FR2) characteristic of camelid antibodies at positions 42, 49, 50 and 52 are indicated with a arrow head [53]. Conventional VH antibodies almost exclusively have V, G, L and W at these positions whereas in camelid VHH's these residues are replaced by 42(F/Y), 49 (E/Q/R), 50(R) and 52 (F/L) which is consistent with the VHH antibodies isolated in this study.(B) SDS-PAGE analysis of purified monovalent VHH antibodies and bivalent VHH antibodies. Molecular weight markers (Mw) 25, 20, 15 kDa.

A phage displayed antibody library was constructed using purified peripheral blood mononuclear cells from the H1 immunised alpaca. The library was selected on immobilised H1-HA (A/California/07/2009) or, alternatively, on recombinant H1-HA followed by H5-HA (A/Vietnam/1203/2004). In all cases a control antibody library selection in the absence of HA was performed so that antigen specific enrichment could be monitored. Primary screening was carried out in a 96 well format with 24 clones being picked from each round of selection and screened for binding to either recombinant H1 (R1a, R2a, R3a) or recombinant H5 (R1a, R2b, R3b). The proportion of antibodies binding to either H1 or H5 increased with consecutive rounds of selection as expected (data not shown). A total of 66 antibody clones were sequenced and grouped on the basis of their unique VHH CDR3 sequence into a total of 18 different antibodies (Figure 1A). There was a significantly greater diversity of antibodies recovered in the H1 selections compared with the alternating H1/H5 selections, with some antibodies being common to both selection strategies. For example, R1a-A5 was a dominant clone (*n = 34/66* clones sequenced) and was retrieved from both the H1 and H1/H5 selection procedures suggesting it was a cross-reacting antibody with high affinity. In addition several groups of clonally related sequences were identified which suggests the presence of antibodies at different stages of affinity maturation (data not shown). All 18 antibodies were found to have characteristic camelid framework 2 substitutions (Figure 1A)[53]. The antibodies R2a-G8, and R1a-E6 had extra cysteine residues in the CDR3 and FR2 in combination with a long CDR3 which is characteristic of some llama single domain antibodies [53], [54]. The sdAbs were, in general, well expressed in the *E.coli* periplasm with yields in shaking flasks of up to 10.6 mg/l of soluble non-aggregating monomeric antibodies (Figure 1B).

### Analysis of influenza virus subtype specificity

**Figure 2:**
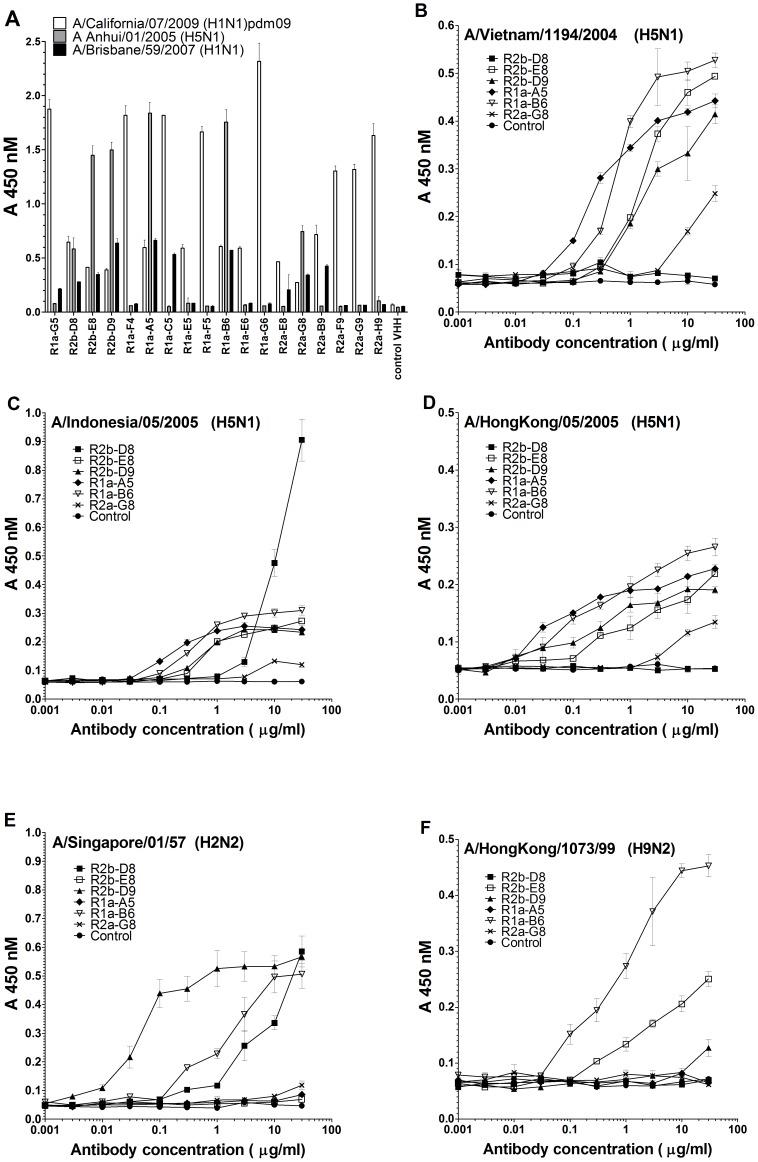
Specificity of single domain antibodies to different influenza antigen reference reagents. ELISA showing binding of 18 purified single domain VHH antibodies to influenza reference reagents prepared from different viral subtypes (A) ELISA comparing binding of purified VHH antibodies at 30 µg/ml against A/California/07/2009 (H1N1pdm09), A/Brisbane/59/2007 (seasonal H1N1),A/Anhui/01/2005 (H5N1) (B) A/Vietnam/1194/2004 (H5N1) (C) A/Indonesia/05/2005 (H5N1) (D) A/HongKong/05/2005 (H5N1) (E) A/Singapore/1/57 (H2N2) (F) A/Hong Kong/1073/99 (H9N2). Antibodies R2b-E8, R2b-D9, R1a-A5, R1a-B6 and R2a-G8 were also positive on A/turkey/Turkey/01/2005 (H5N1), A/Duck/Sing-Q/119-3/97 (H5N3), A/mallard/Eng/727/2006 (H2N3) (data not shown). In addition all antibodies were negative on B virus control (B/Brisbane/60/2008) and on the group 2 strain A/Brisbane/10/07 (H3N2) (data not shown). Control represents a non-binding purified VHH antibody. Binding was measured in duplicate and the average OD 450 nM was plotted against a serial dilution of antibody from 30 µg/ml to 0.001 µg/ml (B-F).

Purified sdAbs were tested in ELISA on a series of different influenza reference reagents (Figure 2A-G). All 18 antibodies were shown to bind to A/California/07/2009 (H1N1)pdm09 and, of these, 10 were shown to also react with the former seasonal A(H1N1) strain A/Brisbane/59/2007. Six antibodies, R2b-D8, R2b-E8, R2b-D9, R1a-A5, R1a-B6, and R2a-G8, were shown to cross-react with different A(H5N1) strains tested (Figure 2A-G). For antibodies R1a-B6, R2b-D9 and R2b-D8 this cross-reactivity could be extended to include the H2 strain A/Singapore/01/57 (H2N2). Antibodies R1a-B6 and R2b-E8 showed cross-reactivity with the H9 strain (A/Hong Kong/1073/99)(H9N2) (Figure 2A-G). All antibodies were negative on A/Brisbane/10/2007 (H3N2) and A/NewYork/107/2003 (H7N2) which belong to phylogenic group 2 of viruses (data not shown).

**Figure 3:**
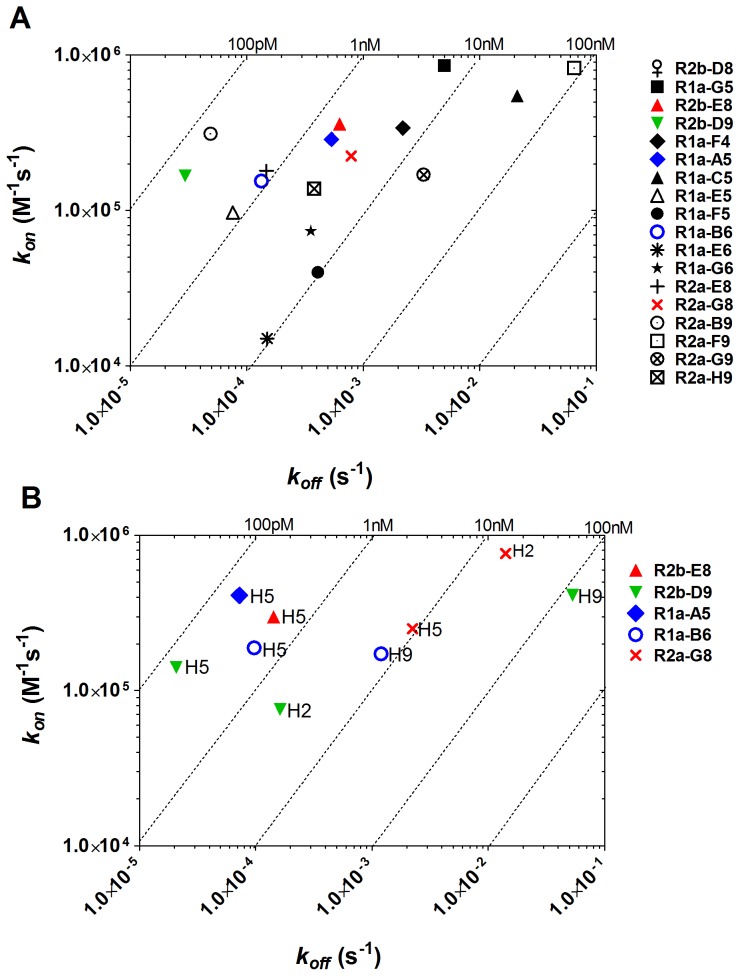
Antibody affinity on recombinant HA by surface plasmon resonance. The kinetic rate binding constants (*kon* and *koff*) of different antibodies were determined using SPR and single cycle kinetics [49]. Data are represented as rate plots with iso-affinity diagonals where the diagonals (dotted lines) are connecting the points of equal dissociation constant (K_D_) ; (A) affinity on recombinant H1-HA, A/California/06/2009 (H1N1pdm09) and (B) affinity of the cross-reactive antibodies on recombinant H5-HA, A/Vietnam/1203/2004 (H5N1) recombinant H2-HA, A/Japan/305/57 (H2N2) and recombinant H9-HA, A/HongKong/1073/99 (H9N2). Fitting was with single cycle kinetics method and a 1∶1 Langmuir fitting model using BIAevaluation software. Equilibrium dissociation constants (K_D_) are given in Table 1.

Analysis of binding specificity and affinity was carried out using surface plasmon resonance (SPR) and single cycle kinetics [49]. The affinity of our panel of sdAbs on recombinant H1 varied from 78.8 nM to 0.18 nM (Table 1, Figure 3A). Further analysis on recombinant HA of other sub-types confirmed that the antibodies R2b-E8, R2b-D9, R1a-A5, R1a-B6, and R2a-G8 were cross-reactive (Table 1, Figure 3B). The affinity on H5-HA was of the same order of magnitude as H1-HA; however, the affinity on HA of more divergent sub-types, H2 and H9, was generally lower (Table 1, Figure 3B). Antibody R2b-D8 although cross-reactive as measured by ELISA on influenza antigen standards did not show any binding to recombinant HA using SPR.

**Table 1:** Specificity and affinity determination using surface plasmon resonance.

	H1-HA (H1N1)			H5-HA (H5N1)			H2-HA (H2N2)			H9-HA (H9N2)		
Clone	*kon* 1 (M^−1^s^−1^)	*koff* (s^−1^)	K_D_ (nM)	*kon* (M^−1^s^−1^)	*koff* (s^−1^)	K_D_ (nM)	*kon* (M^−1^s^−1^)	*koff* (s^−1^)	K_D_ (nM)	*kon* (M^−1^s^−1^)	*koff* (s^−1^)	K_D_ (nM)
**R1a-G5**	8.62×10^5^	5.04×10^−3^	5.85	-	-	-	-	-	-	-	-	-
**R2b-D8**	-	-	−2	-	-	-	-	-	-	-	-	-
**R2b-E8**	3.61×10^5^	6.28×10^−4^	1.74	2.99×10^5^	1.43×10^−4^	0.48	-	-	-	+	+	+^3^
**R2b-D9**	1.67×10^5^	2.96×10^−5^	0.18	1.41×10^5^	2.08×10^−5^	0.15	7.54×10^4^	1.63×10^−4^	2.17	4.13×10^5^	5.28×10^−2^	128
**R1a-F4**	3.40×10^5^	2.20×10^−3^	6.46	-	-	-	-	-	-	-	-	-
**R1a-A5**	2.87×10^5^	5.35×10^−4^	1.87	4.11×10^5^	7.25×10^−5^	0.18	-	-	-	-	-	-
**R1a-C5**	5.48×10^5^	2.06×10^−2^	37.57	-	-	-	-	-	-	-	-	-
**R1a-E5**	9.76×10^4^	7.61×10^−5^	0.79	-	-	-	-	-	-	-	-	-
**R1a-F5**	4.02×10^4^	4.14×10^−4^	10.00	-	-	-	-	-	-	-	-	-
**R1a-B6**	1.55×10^5^	1.34×10^−4^	0.86	1.88×10^5^	9.77×10^−5^	0.52	+	+	+^3^	1.72×10^5^	1.19×10^−3^	6.89
**R1a-E6**	1.51×10^4^	1.51×10^−4^	9.97	-	-	-	-	-	-	-	-	-
**R1a-G6**	7.38×10^4^	3.55×10^−4^	4.83	-	-	-	-	-	-	-	-	-
**R2a-E8**	1.87×10^5^	1.48×10^−4^	0.79	-	-	-	-	-	-	-	-	-
**R2a-G8**	2.25×10^5^	7.98×10^−4^	3.55	2.52×10^5^	2.23×10^−3^	9.07	7.64×10^5^	1.42×10^−2^	18.53	-	-	-
**R2a-B9**	3.12×10^5^	4.94×10^−5^	0.16	-	-	-	-	-	-	-	-	-
**R2a-F9**	8.25×10^5^	6.50×10^−2^	78.80	-	-	-	-	-	-	-	-	-
**R2a-G9**	1.77×10^5^	3.92×10^−3^	22.14	-	-	-	-	-	-	-	-	-
**R2a-H9**	1.39×10^5^	3.84×10^−4^	2.77	-	-	-	-	-	-	-	-	-
**control**	-	-	-	-	-	-	-	-	-	-	-	-

1 Association rate constant *kon*, dissociation rate constant *koff*, equilibrium dissociation constants K_D_ determined by single cycle kinetics on recombinant recombinant H1-HA, A/California/06/2009 (H1N1)pdm09, H5-HA, A/Vietnam/1203/2004 (H5N1), H2-HA, A/Japan/305/57 (H2N2), H9-HA, A/Hong Kong/1073/99 (H9N2). No binding to H3-HA, A/Brisbane/10/2007 (H3N2), H7-HA, A/Netherlands/219/2003 (H7N7) was seen (data not shown).

Specificity and affinity on recombinant H1-HA derived from. Affinity constant K_D_ is given in nM and to 2 decimal places. Control is a non-relevant antibody.

2 - No binding.

3 + Some binding could be detected but was too low to be analysed using BIAevaluation software.

### Evaluation of potency in influenza viral neutralisation assays

**Figure 4:**
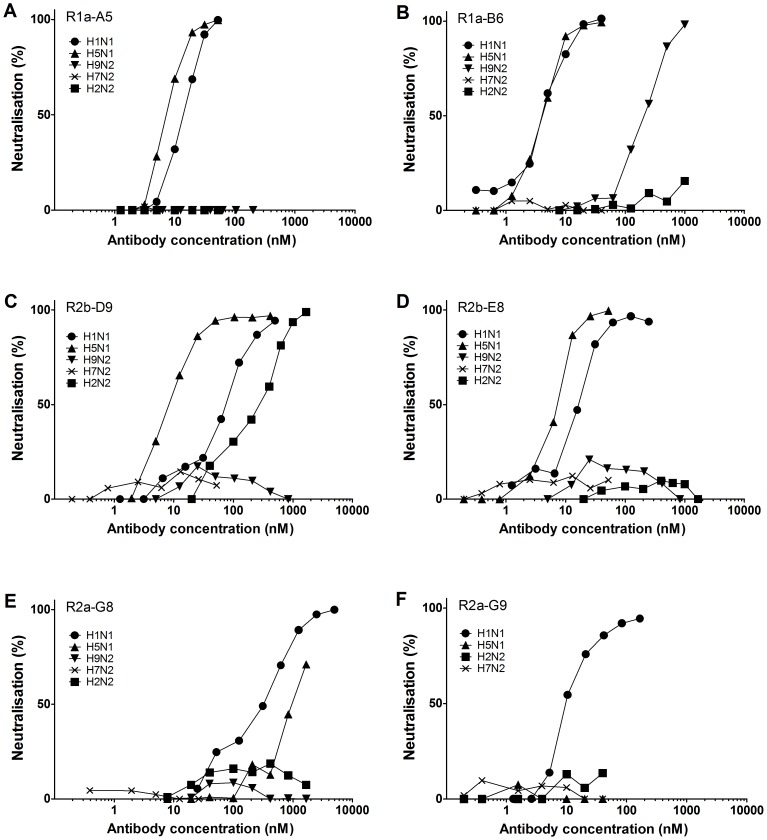
Viral neutralisation activity of cross-reactive sdAbs. Representative curves of five antibodies with cross-neutralising activity (A) R1a-A5 (B) R1a-B6, (C) R2b-D9, (D) R2b-E8, (E) R2a-G8 and an example of an antibody with restricted neutralisation activity (F) R2a-G9. Antibodies were tested for neutralisation activity on laboratory adapted X-181 strain [derived from A/California/07/2009(H1N1)pdm09], NIBRG-14 [reverse genetics reassortant of A/Vietnam/1194/2004 (H5N1)], NIBRG-91 [reverse genetics reassortant of A/chicken/Hong Kong/G9/97(H9N2)], NIBRG-147 [reverse genetics reassortant of A/Singapore/1/57 (H2N2)] and NIBRG-109 [reverse genetics reassortant of A/New York/107/2003 (H7N2)]. Data shown are from one representative neutralisation assay. Data are plotted as the mean of two replicates per assay and antibody concentrations are in nM.

Purified monomeric sdAbs were tested for their ability to neutralise live influenza virus in micro-neutralisation assays. Fifteen of the H1N1 specific antibodies were shown to neutralise X-181 (H1N1) with IC50 values ranging from 3.2 nM to 212.2 nM (Table 2). The antibodies R2b-E8, R2b-D9, R1a-A5, R1a-B6 and R2a-G8 were also shown to cross-neutralise H5N1 (Figure 4). The most potent cross-neutralising sdAbs were R1a-B6 and R1a-A5 with IC50 values in the single digit nanomolar range on both H1N1 and H5N1 (Table 2, Figure 4). The cross-neutralisation activity of R2b-D9 was shown to extend to H2N2 (H1 clade) and for R1a-B6 cross-neutralisation activity was extended to include the more phylogenetically distant virus H9N2 (H9 clade). There was good agreement between binding affinity and neutralisation activity except for R2a-G8 which was shown to be the least potent in neutralisation assays despite having comparable affinity to the other cross-neutralising antibodies.

**Table 2:** Antibody mediated neutralisation of different influenza virus subtypes.

Clone	X-181^1^(H1N1) IC50 nM^2^	NIBRG-14^1^ (H5N1)IC50 nM	NIBRG-147^1^ (H2N2)IC50 nM	NIBRG-91^1^ (H9N2)IC50 nM	NIBRG-109^1^ (H7N2)IC50 nM
**R1a-G5**	14.2±3.1	− ^4^	ND^5^	ND	ND
**R2b-D8**	-	-	ND	ND	ND
**R2b-E8**	40.0±11.4	14.9±4.9	-	-	-
**R2b-D9**	40.7±11.8	14.9±4.7	134.3±67.2	-	-
**R1a-F4**	+^3^	-	ND	ND	ND
**R1a-A5**	11.9±1.3	4.0±2.0	-	-	-
**R1a-C5**	20.2±2.7	-	-	-	-
**R1a-E5**	125.7±7.6	-	ND	ND	ND
**R1a-F5**	-	-	ND	ND	ND
**R1a-B6**	3.2±0.5	5.5±0.9	-	182.2±25.2	-
**R1a-E6**	-	-	ND	ND	ND
**R1a-G6**	7.3±1.1	-	ND	ND	ND
**R2a-E8**	15.0±5.7	-	ND	ND	ND
**R2a-G8**	212.2 ±18.7	>1000	-	-	-
**R2a-B9**	26.3±8.2	-	ND	ND	ND
**R2a-F9**	23.2±6.4	-	ND	ND	ND
**R2a-G9**	17.0±4.2	-	-	ND	-
**R2a-H9**	9.9±2.6	-	ND	ND	ND
**control**	-	-	-	-	-

1 Neutralisation of laboratory adapted X-181 strain corresponding to A/California/07/2009 (H1N1)pdm09, NIBRG-14 a reverse genetics reassortant of A/Vietnam/1194/2004 (H5N1) with the polybasic cleavage site removed from the HA, NIBRG-147 reverse genetics reassortant of A/Singapore/01/57 (H2N2), NIBRG-91 reverse genetics reassortant of A/chicken/Hong Kong/G9/97 (H9N2), NIBRG-109 reverse genetics reassortant of A/New York/107/2003 (H7N7).

2 IC50 is given in nM and is the concentration required to give 50% maximum neutralisation. Data is given as the mean mean of a minimum of three independent assays ± SEM.

3 + indicates neutralisation was seen but a IC50 could not be calculated as a maximum level of neutralisation activity was not reached. ^4^– indicates no neutralisation. ^5^ ND is not determined.

### Characterisation of antibody binding to hemagglutinin and mechanism of viral neutralisation

Hemagglutinin (HA) is the major viral coat protein comprising a trimeric structure of three identical copies of a HA0 pre-cursor polypeptide. HA0 upon cleavage by proteases forms a pH-dependent metastable intermediate composed of HA1 and HA2 subdomains which have distinct roles in the viral infection process [28]. The membrane distal head domain is composed entirely of HA1 residues and contains the receptor binding site which mediates the initial attachment of the virus to sialic acid receptors on host cells [55], [56]. The membrane proximal stem region is assembled from HA2 and some HA1 residues and contains the fusion machinery that undergoes a large conformational change triggered by the low pH environment in the endosomes [28]. Antibodies which neutralise the virus through blocking attachment to cell surface receptors mediated by the globular head domain are identified using a hemagglutination inhibition (HI) assay. Seven sdAbs (R1a-G5, R1a-F4, R1a-C5, R1a-G6, R2a-F9, R2a-G9, R2a-H9) were shown to have HI activity and were confirmed as binding to the HA1 domain using SPR (Table 3). Binding to the HA1 domain and HI activity correlated with antibodies with H1N1 sub-type restricted neutralisation activity which was consistent with these antibodies binding to non-conserved epitopes in the variable HA1 domain.

**Table 3:** Antibody classification.

Clone	Antibody neutralisation category^1^	HI titre (µg/ml) H1N1^2^	H1 (D18-I530)^3^ K_D_ (nM)	HA1 (H1N1) (D18-R344)^3^ K_D_ (nM)	HA^4^ P66-I282	HA^4^ L193-K225	HA^4^ D363-G478	Low pH treated HA 5	Neutral pH treated HA 5
**R1a-G5**	R	1.56	5.85	15.6	+	-	-	+	+
**R2b-D8**	N	-	-	-	-	-	+	++	+
**R2b-E8**	C	-	1.74	-	-	-	-	-	+
**R2b-D9**	C	-	0.18	-	-	-	-	-	+
**R1a-F4**	R	2.08	6.46	4.74	+	-	-	+	+
**R1a-A5**	C	-	1.87	-	-	-	-	-	+
**R1a-C5**	R	10.40	37.57	38.40	+	+	-	+	+
**R1a-E5**	R	-	0.79	-	-	-	-	-	+
**R1a-F5**	N	-	10.00	6.06	+	-	-	+	+
**R1a-B6**	C	-	0.86	-	-	-	-	-	+
**R1a-E6**	N	-	9.97	36.00	+	-	-	+	+
**R1a-G6**	R	1.04	4.83	4.62	+	-	-	+	+
**R2a-E8**	R	-	0.79	-	-	-	-	-	+
**R2a-G8**	C	-	3.55	7.66	-	-	-	-	+
**R2a-B9**	R	-	0.16	-	-	-	-	-	+
**R2a-F9**	R	1.04	78.80	78.80	+	-	-	+	+
**R2a-G9**	R	1.30	22.14	68.70	+	-	-	+	+
**R2a-H9**	R	1.30	2.77	468.00	+	-	-	+	+
**control**	-	-	-	-	-	-	-	-	-

1 Antibody neutralisation category, R is H1N1 restricted neutralisation activity, C refers to a cross-neutralising antibody, N is non-neutralising antibody

2 Hemagglutination inhibition (HI) titre was given as the minimum dilution of antibody at which inhibition of agglutination of turkey erythrocytes was observed (in µg/ml of purified antibody). Values are given as an average of three independent assays. Laboratory adapted A(H1N1) strain (X-181) was used.

3 Single cycle affinity determination on full length HA (D18-I530) and globular head HA1 domain (residues D18-R344) derived from A/California/06/2009 (H1N1pdm09). Affinity is given in nM.

**Figure 5:**
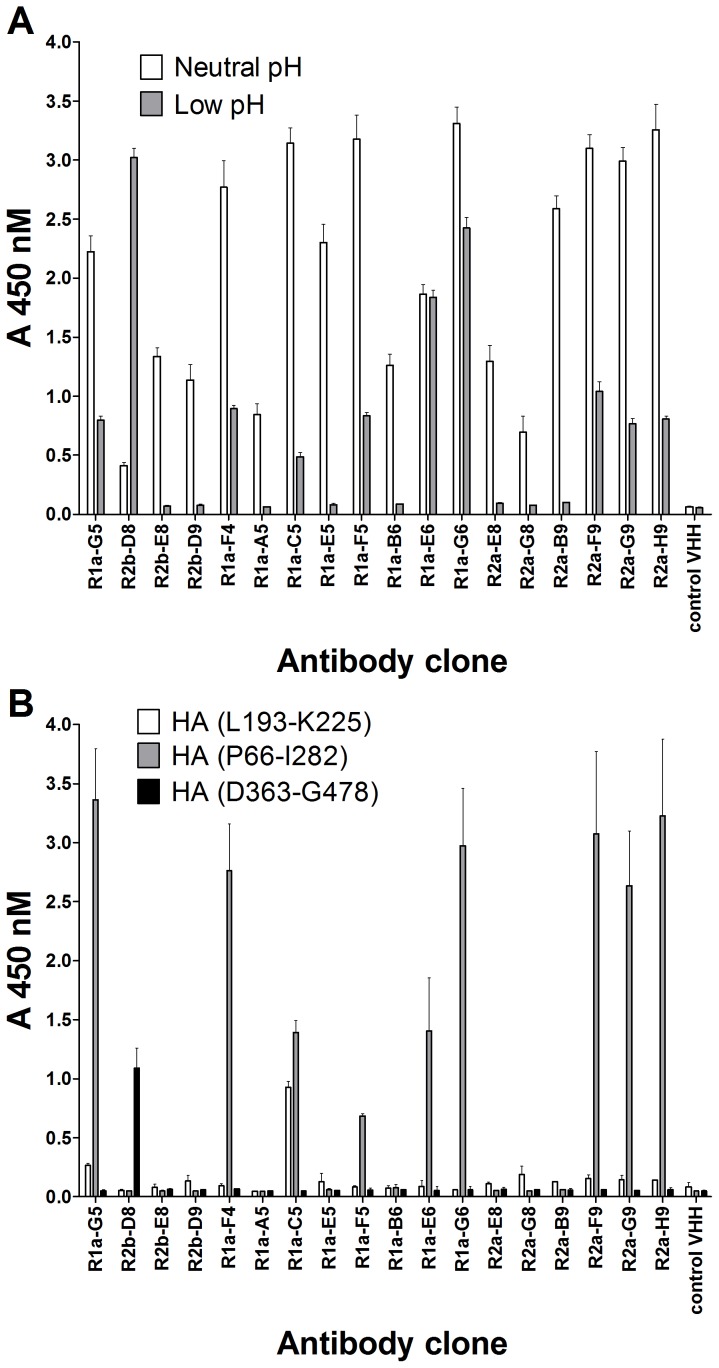
Characterisation of antibody epitopes. (A) ELISA showing reactivity of purified antibodies at 30 µg/ml to HA antigen standard A/California/07/2009 (H1N1)pdm09 either treated with low pH or neutral pH. (B) ELISA showing binding of phage displayed HA gene fragments to purified sdAbs. Antibodies were coated onto an ELISA plate at 10 µg/ml and incubated with purified phage particles. The panel of 18 antibodies were tested for binding to the HA gene fragments L193-K225, P66-I282 and D363-G478 numbered according to Feshchenko *et al*., (2012)[52].

4 Binding to phage displayed fragments of HA as indicated in ELISA (see Figure 5B).

5 ELISA on HA of A/California/07/2009 (H1N1pdm09) treated with low pH and neutral pH (see Figure 5A).

The HI assay, although still considered the ‘gold standard’ serological test to characterise influenza antibodies, is unable to identify cross-neutralising antibodies that bind in the membrane proximal stem region. The over-reliance on this assay has been asserted, by some, as being in part responsible for cross-neutralising antibodies having gone undetected for so long [57]. This is exemplified by the only very recent identification of cross-neutralising human monoclonal antibodies F10 [16] and CR6261 [17]. Both these antibodies are negative for hemagglutination inhibition whereas they show broad activity in viral neutralisation assays. All five cross-neutralising sdAbs identified in this study (R2b-E8, R2b-D9, R1a-B6, R1a-A5 and R2a-G8) were negative in the HI assay suggesting they belong to the same class of cross-neutralising antibodies as F10 [16] and CR6261 [17]. To investigate further which antibodies might interact with the HA stem region we evaluated binding after exposure of HA to low pH. This is predicted to mimic the conformational changes that occur in the stem region during membrane fusion [28]. All sdAbs with cross-neutralisation activity lost binding to H1N1 after low-pH treatment which was consistent with their binding to pH-sensitive epitopes in the stem region. Conversely all sdAbs with HI activity were shown to retain binding after low-pH treatment which is in agreement with their binding to, or in the vicinity of, the sialic acid receptor binding site in the membrane distal head domain (Figure 5A). The antibody R2a-G8 appeared distinct from the other cross-neutralising antibodies in that it only had weak cross-neutralising activity (Table 2) despite having comparable affinity (Table 1). In addition, although R2a-G8 lost binding after low pH treatment and was negative for HI activity it was still able to bind to purified HA1 domain in SPR (Table 3) further exemplifying the unique characteristics of this antibody in our panel. The antibody R2b-D8 also had distinct characteristics in that although it showed cross sub-type reactivity it did not cross-neutralise the virus and surprisingly showed an increased binding to HA upon low pH treatment (Figure 5A). This suggests that R2b-D8 recognises an epitope which is exposed when HA goes through low pH induced conformational change.

A phage displayed library of a fragmented H1-HA gene derived from A/California/07/2009 (H1N1)pdm09 was constructed in order to further refine epitope characteristics of our antibody panel. After two rounds of selection on immobilised R1a-C5 over 50% of the clones analysed corresponded to either HA fragment (P66-I282) or the overlapping HA fragment (L193-K225). Both phage displayed HA fragments mapped to the globular head HA1 domain and binding to R1a-C5 was confirmed in phage ELISA. Subsequently all 18 antibodies were tested for binding to these HA1 domain gene fragments and revealed that antibodies that were positive for HI activity and retained binding after low pH treatment (i.e. R1a-G5, R1a-F4, R1a-C5, R1a-F5, R1a-G6, R2a-F9, R2a-G9, R2a-H9) also showed binding to the HA1 domain gene fragment HA (P66-I282)(Figure 5B, Table 3). None of the cross-neutralising sdAbs showed any binding to these HA1 domain gene fragments. Library selection with the cross-reactive antibodies R1a-B6 and R2b-D9 did not recover any phage binding clones and only out of frame sequences were retrieved (data not shown). In order to isolate a larger collection of phage displayed HA gene fragments library selections were carried out on polyclonal serum from sheep hyper-immunised with A(H1N1)pdm09. This resulted in a much greater diversity of phage displayed fragments which covered epitopes in both the HA1 domain and the HA2 domain including a phage displayed HA2 fragment corresponding to D363-G478. Subsequent testing showed antibody R2b-D8 was able to bind to D363-G478 gene fragment (Figure 5B).

**Figure 6:**
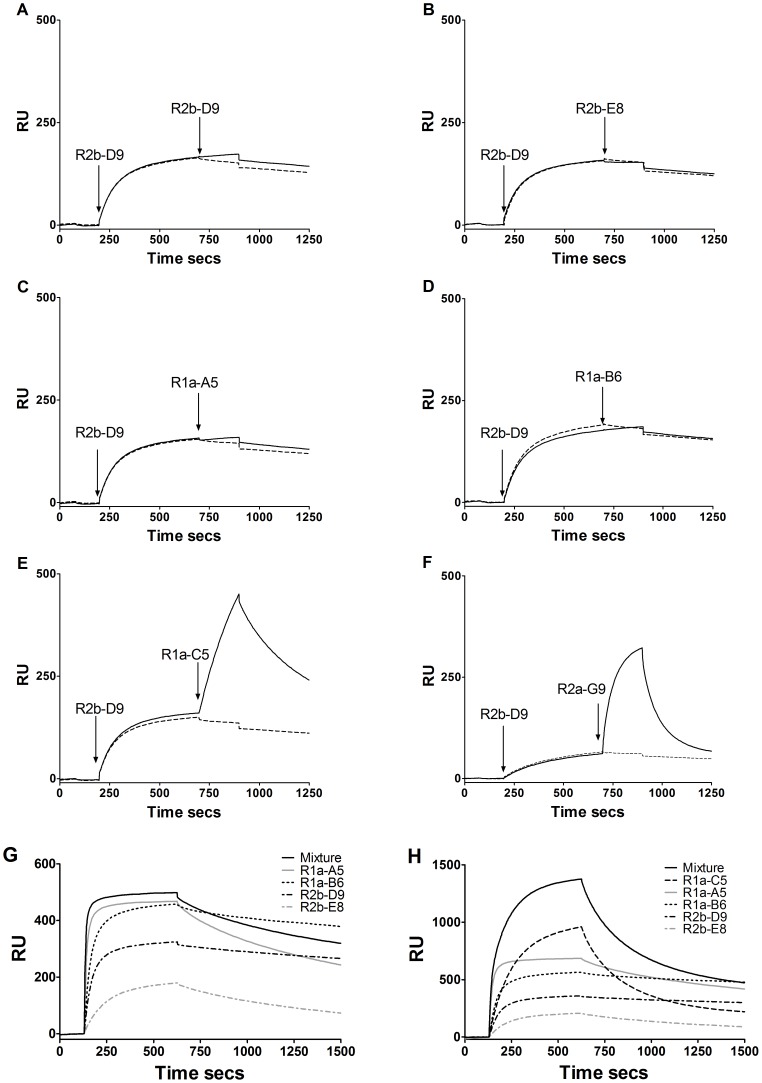
Grouping of antibody epitopes. SPR co-injection experiments were used to determine if pairs of VHH antibodies can bind to recombinant H1-HA simultaneously. The antibodies R1a-A5, R1a-B6, R2b-D9, R2b-E8, R1a-C5 and R2a-G9 were tested in combination with each other. Example series of sensorgrams of R2b-D9 injected as the first antibody followed by injection of each of the other antibodies as indicated (A) R2b-D9/R2b-D9 (B) R2b-D9/R2b-E8 (C) R2b-D9/R1a-A5 (D) R2b-D9/R1a-B6 (E)R2b-D9/R1a-C5 (F) R2b-D9/R2a-G9. The solid line represents co-injection of a first antibody and then a second antibody whereas a dotted line represents injection of a first antibody followed by injection of buffer. Antibodies R1a-B6, R1a-A5, R2b-D9, and R2b-E8 appeared to share overlapping epitopes as no significant increase in response was observed following injection of a second antibody. The antibodies R1a-C5 and R2a-G9 bound a distinct non-overlapping epitope as a significant increase in response was seen upon binding of these antibodies as the second antibody species. The four antibodies R2b-D9, R1a-B6, R2b-E8 and R1a-A5 predicted to share overlapping epitopes were further analysed by injecting each VHH on their own or as equimolar mixture of all four antibodies (Mixture) (G). The sensorgrams indicate the Rmax value for each of the individual antibodies (∼150 RU's to 450 RU's) with no significant increase in Rmax following injection of the antibody mixture, which suggests these antibodies recognise an overlapping epitope or an epitope that hinders binding of a second antibody. If the antibodies recognised non-overlapping or non-hindering sites the Rmax would be expected to be the sum of the individual Rmax values. This was confirmed when R1a-C5 which was predicted to recognise a non-overlapping epitope was included in a equimolar mixture of five antibodies and the response was seen to increase by an amount equivalent to R1a-C5 binding individually (H). Analysis of R2a-G8 was not conclusive.

The cross-neutralising sdAbs were tested to determine whether or not they bound to overlapping or non-overlapping epitopes on H1-HA. SPR co-injection experiments were carried out with pairs of the cross-neutralising VHH antibodies and with the HA1 head specific antibodies R1a-C5 and R2a-G9 to determine whether or not antibodies could bind simultaneously to H1-HA (Figure 6). Four of the cross-neutralising antibodies (R1a-B6, R2b-E8, R2b-D9 and R1a-A5) were shown to bind overlapping epitopes as no significant increase in binding was seen when these antibodies were tested in combination. By comparison, the head-binding antibodies R1a-C5 and R2a-G9, recognised non-overlapping epitopes as they showed significant binding when sdAbs R2b-E8, R1a-B6, R2b-D9, and R1a-A5 were already bound to H1-HA (Figure 6). This was confirmed by demonstrating that there was no increase in response levels for an equimolar mixture of the four cross-neutralising antibodies compared with the individual antibodies (Figure 6G).

**Figure 7:**
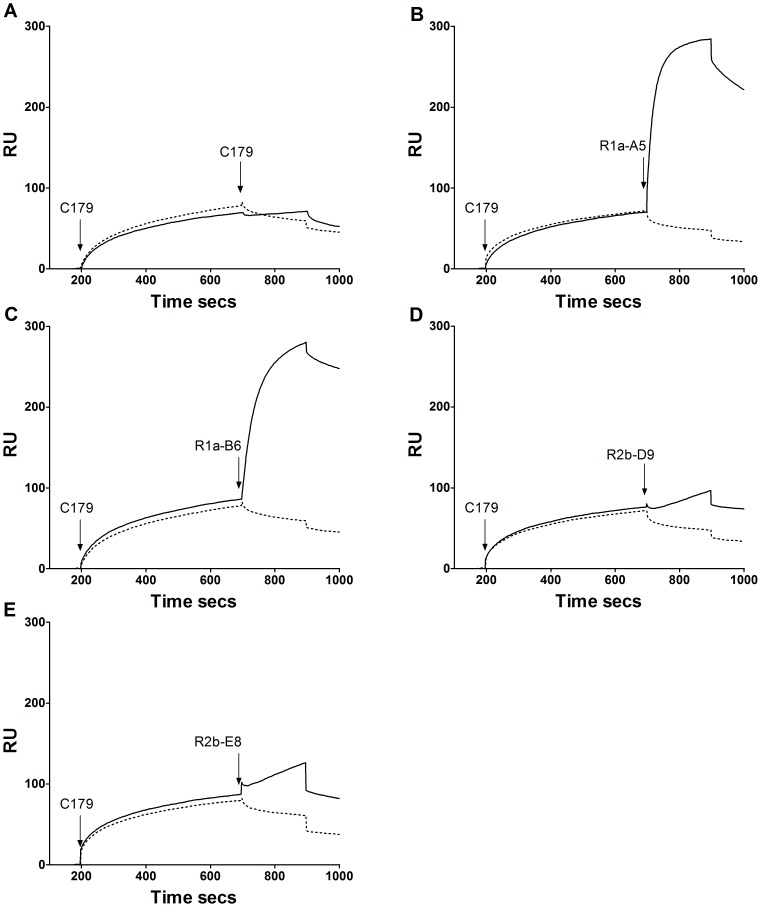
Evaluation of competition with C179. SPR co-injection experiments were used to determine if the cross-neutralising antibodies R1a-B6, R1a-A5, R2b-D9 and R2b-E8 competed with the cross-neutralising mouse monoclonal antibody C179 shown to bind to the HA stem region [50], [51]. The antibody C179 was injected first and the test VHH antibody injected as the second antibody species (A) C179/C179 (B) C179/R1a-A5 (C) C179/ R1a-B6, (D) C179/R2b-D9 and (E) C179/R2b-E8. The solid line represents co-injection of a first antibody and then a second antibody whereas a dotted line represents injection of a first antibody followed by injection of buffer. Analysis of R2a-G8 was not conclusive.

In similar SPR co-injection experiments antibodies R1a-B6, R1a-A5, R2b-D9 and R2b-E8 were tested for competition with the well characterised murine monoclonal antibody C179 which has previously been shown to neutralise H1, H2 and H5 virus subtypes [50] [51]. The epitope for C179 has been mapped to the HA stem region and shown to block membrane fusion rather than cell attachment. Both R2b-D9 and R2b-E8 did not show any further appreciable binding to H1-HA when injected after C179 was injected as the first antibody. In contrast the antibodies R1a-A5 and R1a-B6 were able to bind when C179 was already bound to HA (Figure 7). This suggests that R2b-D9 and R2b-E8 have partial overlapping epitopes with C179 whereas R1a-B6 and R1a-A5 bind to epitopes distinct from C179.

### Evaluation of the cross-neutralising antibodies R1a-B6 and R1a-A5 in a bivalent format

**Figure 8:**
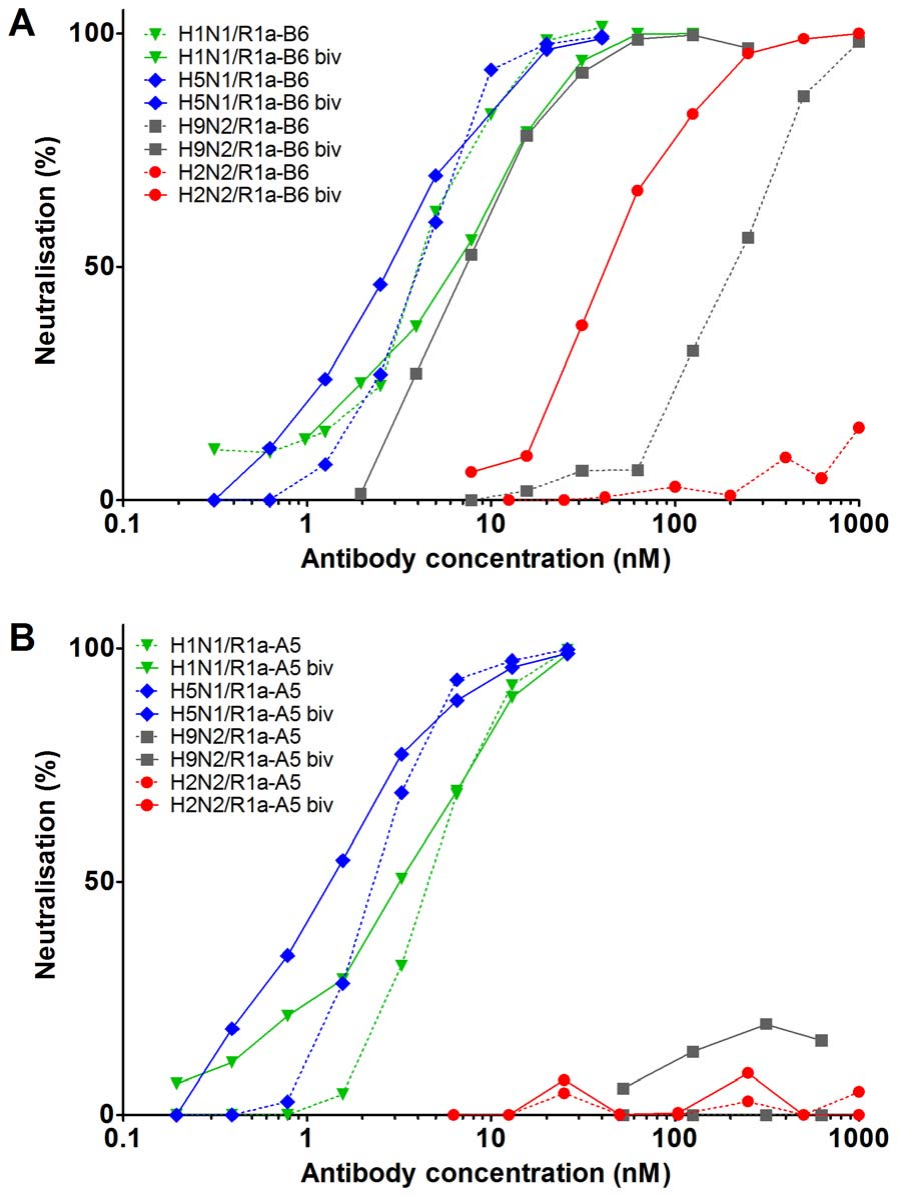
Comparison of viral neutralisation activity with monovalent and bivalent antibody formats. Monovalent and bivalent versions of R1a-B6 (A) and R1a-A5 (B) were compared in neutralisation assays with laboratory adapted X-181 strain [corresponding to A/California/07/2009(H1N1)pdm09], NIBRG-14 [reverse genetics reassortant of A/Vietnam/1194/2004(H5N1)], NIBRG-91 [reverse genetics reassortant of A/chicken/Hong Kong/G9/97(H9N2)], NIBRG-109 [reverse genetics reassortant of A/New York/107/03(H7N2)] and NIBRG-147 [reverse genetics reassortant of A/Singapore/1/57(H2N2)]. Representative curves are shown and are the mean of two replicates. Antibody concentrations are in nM.

Avidity has been shown to significantly increase the potency of both human antibodies [58]–[60] and single domain antibodies [61] which neutralise by blocking the receptor binding site in the HA1 domain. We investigated whether similar increases in potency were possible with cross-neutralising antibodies which neutralise virus by post-cell attachment mechanisms. As such bivalent versions of R1a-B6 and R1a-A5 were produced by fusing two identical binding domains separated by a thirty amino acid glycine linker. We did not observe any significant increase in potency on X-181 (H1N1) or NIBRG-14 (H5N1) for either bivalent antibody (Table 4, Figure 8). However, surprisingly we observed that bivalent R1a-B6 had gained the ability to neutralise H2N2 (NIBRG-147) and showed a substantial increase in potency against the more divergent strain H9N2 (NIBRG-91). In the case of monovalent R1a-B6 no neutralisation activity could be detected on NIBRG-147 (H2N2) down to 1 µM antibody concentration whereas bivalent R1a-B6 had an IC50 of 36.6 nM, representing an increase in potency of at least 100-fold. Similarly, at least a 17 fold increase in potency was observed for the bivalent R1a-B6 on NIBRG-91 (H9N2) (Table 4, Figure 8). In addition we compared the apparent affinity of monovalent and bivalent sdAbs using SPR on high density surfaces of recombinant H1, H5, H2, H9, H3 and H7-HA. We observed significant increases in apparent affinity on more divergent viral subtypes which was due largely to improvement in antibody off rates (Figure S1, Table S2). This was consistent with the second VHH domain in our tandem linked bivalent antibodies being able to functionally interact with HA.

**Table 4:** Comparison of viral neutralisation activity of R1a-B6 and R1a-A5 in a monovalent and bivalent antibody format.

Clone	X-181^1^ (H1N1) IC50 nM^2^	NIBRG-14^1^ (H5N1) IC50 nM	NIBRG-147^1^ (H2N2) IC50 nM	NIBRG-91^1^ (H9N2) IC50 nM	NIBRG-109^1^(H7N2) IC50 nM	Seasonal^1^ (H3N2) IC50 nM
**R1a-B6 monovalent**	3.2±0.5	5.5±0.9	-	182.2±25.2	−3	-
**R1a-B6 bivalent**	9.4±3.2	2.4±0.3	36.6±5.7	10.2±2.9	-	-
**R1a-A5 monovalent**	11.9±1.3	4.0±2.0	-	-	-	NT
**R1a-A5 bivalent**	6.7±1.0	1.8±0.2	-	-	NT	NT
**control**	-	-	-	-	-	-

1 Neutralisation of laboratory adapted X-181 strain corresponding to A/California/07/2009 (H1N1)pdm09, NIBRG-14, a reverse genetics reassortant of A/Vietnam/1194/2004 (H5N1), NIBRG-147, a reverse genetics reassortant of A/Singapore/01/57 (H2N2), NIBRG-91 a reverse genetics reassortant of A/chicken/Hong Kong/G9/97 (H9N2), NIBRG-109 reverse genetics reassortant of A/New York/107/2003 (H7N2) and seasonal A(H3N2) virus A/Montana/05/2011.

2 IC50 is given in nM and is the concentration required to give 50% maximum neutralisation. Results are the mean of 3 or more independent assays.

3 – indicates no neutralisation was seen at antibody concentrations less than 1 µM.

NT is not tested.

## Discussion

An increasing number of cross sub-type neutralising monoclonal antibodies have been identified which target conserved regions of the HA stem region [16]–[23], [32]. All of these antibodies require a paired light chain and heavy chain to form a stable folded antibody capable of antigen interaction. However elucidation of the structure of two antibodies F10, [16] and CR6261 [17], in complex with HA has shown that only the heavy chain makes antigen contact with the light chain being superfluous to requirements. With this in mind we have chosen to use sdAbs derived from alpacas as a route to high affinity cross-neutralising antibodies which are naturally devoid of a paired light chain. In addition, the tendency to have long CDR3 loops has suggested that this unique antibody format may be well equipped to access cryptic epitopes on viral particles [40], [62]. A further potential advantage of this antibody format is their smaller antigenic footprint compared with conventional two chain human antibodies which have evolved to bind to larger flatter surfaces. It is interesting to speculate that viral escape from single domain antibodies could be more difficult than from conventional two chain monoclonal antibodies [63], [64].

We have identified 18 alpaca sdAbs specific for pandemic influenza 2009 A(H1N1) which have been grouped as having H1N1 subtype restricted neutralising activity, non-neutralising activity, or broad cross-subtype neutralising activity (Table 3). All antibodies with HI activity (i.e R1a-G5, R1a-F4, R1a-C5, R1a-G6, R2a-F9, R2a-G9 and R2a-H9) were shown to belong to the group having H1N1 sub-type restricted neutralisation activity. In addition this group showed binding to recombinant HA1 domain and retained binding to HA after low-pH treatment (Table 3). These data are consistent with binding to epitopes at or near to the receptor binding site in the HA1 domain. For one of the antibodies belonging to this group, R1a-C5, the epitope was located to residues L193-K225 in the HA1 domain (Figure 5B). We note that structural analysis has shown this region to span helix 190 of the sialic acid receptor binding site [65] which correlates with the proposed mechanism of action of R1a-C5.

The three non-neutralising antibodies R1a-F5, R1a-E6 and R2b-D8 belonged to two distinct groups. The antibodies R1a-F5 and R1a-E6 were negative for HI activity; however, they bound to the HA1 domain and retained binding to HA after low pH treatment. This suggests that these antibodies still bind to epitopes within the HA1 domain but outside of the sialic acid receptor binding site or epitopes that can impact viral binding to sialic acid receptors on the cell surface (Table 3). The other non-neutralising antibody R2b-D8 was unique amongst our panel of antibodies in that it showed cross-reactivity against influenza antigen standards but did not bind to recombinant HA in SPR nor was it capable of virus neutralisation (Table 3). This suggests that R2b-D8 binds to a non-native epitope on HA. In addition, the increase in binding to HA at low pH suggests that this epitope is in the stem region. This conclusion was supported by the binding of R2b-D8 to the phage displayed HA stem region fragment (D363-G478)(Figure 5B). Although it may seem surprising that R1a-A5, R1a-B6, R2b-D9 and R2b-E8 did not bind to the HA stem fragment D363-G478 we think this simply reflects these antibodies being distinct from R2b-D8 in that they recognise a functionally relevant epitope which is not formed when the D363-G478 fragment is isolated from the rest of the HA molecule. This correlates with previous reports that only the low pH induced conformation can be formed when the HA2 domain is expressed in *E.coli* in the absence of the globular head HA1 domain [66].

Of perhaps the greatest interest are the five cross-neutralising antibodies, the most potent of which being R1a-B6 which had IC50 values on X-181 (H1N1) and NIBRG-14 (H5N1) in the single digit nanomolar range (Table 2). This is comparable with the cross-neutralising antibody CR6261 [17] which has a IC50 of 3–4 nM on NIBRG-14 (H5N1) as an IgG. Our data indicates that the cross-neutralising antibodies belong to two separate groups with R2a-G8 being distinct from the other four antibodies. The cross-neutralising antibodies (R2b-E8, R2b-D9, R1a-A5 and R1a-B6) were all shown to bind to full length HA but not to the HA1 domain and in addition lost binding to HA at low pH. This suggests that these antibodies bind to epitopes in the membrane proximal stem region away from the receptor binding site [16], [17]. The mechanism of action of the cross-neutralising human monoclonal antibodies F10 [16] and CR6261 [17], [28] have been shown to be mediated through blocking the low pH induced conformational changes in the membrane proximal stem region which mediates membrane fusion [28]. The loss of binding of the cross-neutralising antibodies R2b-D9, R2b-E8, R1a-A5, R1a-B6 and R2a-G8 following low-pH treatment of HA is conducive with these antibodies using similar post viral attachment mechanisms to neutralise virus.

Antibody R2a-G8 was distinct from the other four cross-neutralising antibodies in that there was a much lower neutralisation activity than the binding affinity would predict. In addition, despite the absence of HI activity it showed strong binding to purified HA1 domain (D18-R344). The absence of binding to the smaller HA fragment (P66-I282) (Table 3, Figure 5B) implies that the epitope for R2a-G8 lies either at the extreme N-terminus (residues 18–65) or extreme C-terminus (residues 283–344) of the HA1 domain. As our data suggest that R2a-G8 binds to a part of the HA1 domain that lies within the stem region it is interesting to note that structural data place both the extreme N-terminal residues (18–65) and extreme C-terminal residues (283–344) of HA1 within this region and away from the globular head [28].

Whether monoclonal antibodies are able to bind with high avidity to HA is dependent on multiple parameters including accessibility, orientation and density of the specific epitopes on the viral surface as well as properties intrinsic to the antibody molecule itself, such as, size, flexibility and affinity. Generally, subtype specific antibodies targeting the receptor binding site in the HA1 domain, have a significantly greater potency as an IgG relative to a corresponding monovalent antibody fragment [58], [59], [61]. This substantial enhancement in potency is due to either cross-linking adjacent HA trimeric spikes on a single virion or through the aggregation of separate viral particles. In the case of cross-neutralising antibodies which bind to the less accessible membrane proximal stem region it is not clear to what extent avidity may play a role if at all. For example, the recently described human cross-neutralising antibody CR6261, which binds to a highly conserved epitope in the HA stem, was reported as having similar binding and neutralising activity as both a monovalent Fab fragment and a bivalent IgG [17]. The exact reasons for this are not clear but are likely related to the mechanism of action, epitope accessibility, antibody size or steric constraints.

As the cross-neutralising sdAbs described in this study are a fraction of the size of conventional human monoclonal antibodies we reasoned that they may have a greater potential to utilise avidity. To explore this possibility we converted two of the cross-neutralising antibodies, R1a-B6 and R1a-A5, into a bivalent format but did not see any significant increase in neutralisation activity on H1 and H5 viruses for either antibody. However we saw a substantial increase in potency of bivalent R1a-B6 on the more divergent H2 and H9 subtype viruses. Rather than increasing maximum attainable levels of potency our data shows that conversion of R1a-B6 into a bivalent format increases the breadth of cross-neutralisation activity. This is likely due to a slower off rate which rescues weaker affinity interactions of the monovalent format.

Our data support a distinction in the relationship between avidity and the mechanism of action of neutralising antibodies to HA. For antibodies which function by inhibiting viral attachment to cells, avidity has been shown to significantly increase potency by several orders of magnitude likely through the aggregation or cross-linking of viral particles [58], [59], [61]. However, for cross-neutralising antibodies such as R1a-A5, R1a-B6 and CR6261 [17] that inhibit post-viral attachment steps in the infection process, aggregation or cross-linking of viral particles appear to have a minimal impact on the maximum levels of potency. We suggest that this is due to the antibody mediating its effect after the virus has already attached to the cell surface and been internalized, which implies that the rate of viral internalisation may be a rate limiting step in the activity of stem binding antibodies. We speculate that both monovalent R1a-B6 and R1a-A5 may have reached a limit of activity in neutralisation assays for A(H1N1). Consequently, bivalency does not have the opportunity to enhance potency any further due to the uptake of antibody being restricted by the rate of viral internalization. For lower affinity interactions with A(H2N2) and A(H9N2) we propose that monovalent R1a-B6 is also limited by virus internalisation, except in this case the antibody binding to the virus on the cell surface is insufficient to support efficient antibody uptake and neutralisation activity. In this scenario avidity is able to rescue the weaker affinity interactions of monovalent R1a-B6 to more divergent viral sub-types, so enhancing binding to cell surface virus, boosting antibody internalisation and ultimately increasing potency in viral neutralisation assays.

Single domain antibodies from camelids have shown themselves to be capable of potent neutralisation of a range of different viruses including HIV-1 [67], rotavirus [68], [69], hepatitis B [70] and influenza virus [61], [71]. Such antibodies to pandemic influenza viruses could be expected to have considerable potential for the treatment of pandemic influenza. The recent report of a Nanobody, Infl-C8, and the demonstration of protection in mice against H5N1 following intra-nasal delivery validates that this unique type of antibody has clear potential as an anti-viral agent [61], [71]. All of the antibodies, including Infl-C8, described by Hultberg *et al* (2011) bind to the sialic acid receptor binding site in the HA1 domain, have HI activity and have neutralisation activity limited to H5N1. Antibody Infl-C8 has an IC50 of 7nM on NIBRG-14 which is comparable to the cross-neutralising antibodies in this study (Table 3). Conversion of Infl-C8 into a bivalent format was reported to enhance neutralisation activity on H5N1 by over 1000-fold which reflects the different mechanism of action of Infl-C8 compared with the cross-neutralising antibodies R1a-A5 and R1a-B6 described in this study. Whilst we have identified equivalent antibodies with restricted neutralising activity limited to pandemic 2009 A(H1N1), we have, to our knowledge, isolated the first sdAbs with cross-subtype neutralisation activity. The influenza virus is constantly changing and sdAbs with broad neutralising activity against divergent influenza subtypes of pandemic potential (i.e H1, H2, H5, H9) could be expected to have significant potential in assisting in preparation for the next pandemic emergency.

The simple structure and high intrinsic stability of camelid single domain antibodies [72], [73] offers the possibility of both intra-nasal delivery [71] and systemic delivery since current antibody engineering technology can facilitate both humanisation [74], serum half–life extension [39] and incorporation of effector function [75]. In addition, their simple conversion into multi-specific formats offers greater flexibility in construction of antibodies targeting different epitopes. The study of the antibody structure in complex with HA will identify if there are any distinct features in the mode of antigen recognition compared with equivalent human monoclonal antibodies which may aid the design of a universal vaccine. The potential applications of cross-reactive single domain antibodies to pandemic influenza extends beyond immune prophylaxis and includes vaccine standardisation [76], serological surveillance [77], vaccine design [9]–[11], and virus purification.

## Supporting Information

Figure S1 **Comparison of apparent affinities of bivalent R1a-B6 and R1a-A5 using surface plasmon resonance.** Apparent affinity of bivalent (biv) and monovalent (mono) versions of R1a-B6 and R1a-A5 were compared using SPR and single cycle kinetics [49] on high density surface surfaces (approximately 10,000 RU) of recombinant H1, H5, H2 and H9. Data are represented as rate plots with iso-affinity diagonals where the diagonals (dotted lines) are connecting the points of equal dissociation constant (K_D_). Fitting was with single cycle kinetics method and a 1∶1 fitting model using BIAevaluation software. Only weak binding of R1a-B6 monovalent to H2-HA could be seen and could not be analysed using BIAevaluation software. No binding on H3 or H7-HA was observed.
(TIF)

Table S1 **Assessment of the serological immune response in immunised alpacas.**
(DOCX)

Table S2 **Comparison of the binding kinetics of bivalent verses monovalent cross-neutralising antibodies R1a-B6 and R1a-A5 on different hemagglutinin subtypes.**
(DOCX)
