# Supplementary material for: The Breadth of Cross Sub-Type Neutralisation Activity of a Single Domain Antibody to Influenza Hemagglutinin Can Be Increased by Antibody Valency
Source: PLoS One. 2014 Aug 1;9(8):e103294. doi: 10.1371/journal.pone.0103294 (PMC4118869; doi:10.1371/journal.pone.0103294)
Supplement: Table S1 — Assessment of the serological immune response in immunised alpacas. (DOCX) [file pone.0103294.s002.docx]

**Table S1.** Assessment of the serological immune response in immunised alpacas.

| **Immunisation strategy** | | | | **HI titre^2^** | | | | | **MN titre^3^** |
| --- | --- | --- | --- | --- | --- | --- | --- | --- | --- |
| **Antigen** | **Immunization** | **Bleed** | **Day** | **A/California/07/2009(H1N1)pdm09** | **B/Brisbane/60/2008Control** | **A/Uruguay/716/2007(H3N2)** | **A/HongKong/213/2003(H5N1)** | **A/Vietnam/1194/2004(H5N1)** | **A/California/07/2009(H1N1)pdm09** |
|  | Primary | Pre - immune | 0 | <8 | <8 | <8 | <8 | <8 | <10 |
| H1 ^1^ | Boost 1 | First bleed | 21 | 64 | <8 | <8 | ND^4^ | ND | ND |
| H1 | Boost 2 | Second bleed | 43 | 2048 | <8 | <8 | ND | ND | ND |
| H1 | Boost 3 | Third bleed | 71 | 8192 | <8 | <8 | <8 | <8 | >1280 |

^1^ H1, purified recombinant HA-H1derived from influenza strain A/California/07/2009 (H1N1)pdm09.

^2^HI titre, hemagglutination inhibition assay and titres are indicated as the reciprocal of the minimum serum dilution at which inhibition of agglutination of turkey erythrocytes is observed using the strains indicated.

^3^MN titre, microneutralisation titre is given as the reciprocal of the minimum dilution at which inhibition of lysis of MDCK cells by influenza strain A/California/07/2009 (H1N1)pdm09 in ELISA.

^4^ND is not determined.
