# Supplementary material for: The Breadth of Cross Sub-Type Neutralisation Activity of a Single Domain Antibody to Influenza Hemagglutinin Can Be Increased by Antibody Valency
Source: PLoS One. 2014 Aug 1;9(8):e103294. doi: 10.1371/journal.pone.0103294 (PMC4118869; doi:10.1371/journal.pone.0103294)
Supplement: Table S2 — Comparison of the binding kinetics of bivalent verses monovalent cross-neutralising antibodies R1a-B6 and R1a-A5 on different hemagglutinin subtypes. (DOCX) [file pone.0103294.s003.docx]

**Table S2.** Comparison of the binding kinetics of bivalent versus monovalent cross-neutralising antibodies R1a-B6 and R1a-A5 on different hemagglutinin subtypes.

|  | **H1-HA** | | | **H5-HA** | | | **H9-HA** | | | **H2-HA** | | |
| --- | --- | --- | --- | --- | --- | --- | --- | --- | --- | --- | --- | --- |
|  | ***kon***  **(M^-1^s^-1^)** | ***koff***  **(s^-1^)** | **K_D_**  **nM** | ***kon***  **(M^-1^s^-1^)** | ***koff***  **(s^-1^)** | **K_D_**  **nM** | ***kon***  **(M^-1^s^-1^)** | ***koff***  **(s^-1^)** | **K_D_**  **nM** | ***kon***  **(M^-1^s^-1^)** | ***koff***  **(s^-1^)** | **K_D_**  **nM** |
| R1a-A5 monovalent | 4.41x10^5^ | 6.85x10^-4^ | 1.56 | 6.22x10^5^ | 1.68x10^-4^ | 0.27 | - | - | - | - | - | - |
| R1a-A5 bivalent | 6.53x10^5^ | 2.19x10^-4^ | 0.36 | 7.06x10^5^ | 1.67x10^-4^ | 0.24 | - | - | - | - | - | - |
| R1a-B6 monovalent | 3.70x10^5^ | 1.32x10^-4^ | 0.36 | 2.57x10^5^ | 1.32x10^-4^ | 0.51 | 3.12x10^5^ | 1.31x10^-3^ | 4.19 | + | + | + |
| R1a-B6 bivalent | 2.67x10^5^ | 3.40x10^-5^ | 0.13 | 1.50x10^5^ | 4.00x10^-6^ | 0.031^a^ | 1.28x10^5^ | 3.00x10^-5^ | 0.23 | 2.02x10^5^ | 2.60x10^-4^ | 1.29 |

Association rate constant *kon*, dissociation rate constant *koff* , equilibrium dissociation constants K_D_ determined by single cycle kinetics on a high density surface (approximately 10,000 RU) of recombinant H1, H5, H9 and H2-HAs. No binding on H3 or H7-HA was observed.

^a^ indicates binding constants were beyond the limits that could be accurately determined by the instrument.

+ binding could be seen but the kinetic data could not be analysed using the BIAevaluation software

- represents no binding seen.
